# A comprehensive review of experimental models and induction protocols for avian necrotic enteritis over the past 2 decades

**DOI:** 10.3389/fvets.2024.1429637

**Published:** 2024-07-24

**Authors:** Mohammad Ali Shamshirgaran, Mehdi Golchin

**Affiliations:** Department of Pathobiology, Faculty of Veterinary Medicine, Shahid Bahonar University of Kerman, Kerman, Iran

**Keywords:** necrotic enteritis, *Clostridium perfringens*, disease induction, experimental challenge, predisposing factors, poultry

## Abstract

Necrotic enteritis (NE) is a severe gastrointestinal disease that poses a significant threat to the poultry industry. It leads to progressive damage to the small intestine, reduced performance, increased mortality rates, and substantial economic losses. With the removal of antimicrobial agents from chicken feed, there is an urgent need to find alternative approaches for NE control. Various approaches, including vaccination, prebiotics, probiotics, and plant-derived products, have been utilized to address NE in poultry management. To evaluate the efficacy of these preventive measures against NE, successful induction of NE is crucial to observe effects of these approaches in related studies. This study presents a comprehensive overview of the methods and approaches utilized for NE reproduction in related studies from 2004 to 2023. These considerations are the careful selection of a virulent *Clostridium perfringens* strain, preparation of challenge inoculum, choice of time and the route for challenge inoculum administration, and utilization of one or more predisposing factors to increase the rate of NE occurrence in birds under experiment. We also reviewed the different systems used for lesion scoring of NE-challenged birds. By gaining clarity on these fundamental parameters, researchers can make informed decisions regarding the selection of the most appropriate NE experimental design in their respective studies.

## 1 Introduction

Necrotic enteritis (NE) is a highly prevalent clostridial enteropathogenic ailment in poultry, with its initial documentation dating back to the 1950s in broiler chickens (1). NE poses a substantial challenge to the advancement of the poultry industry, particularly in nations with significant poultry production (2). It is widely recognized as a prominent limitation hindering the development and expansion of the poultry sector (2, 3). Based on estimations, NE disease outbreaks have had a considerable economic impact, reaching approximately $2 billion in 2000 and escalating to around $6 billion in 2015, corresponding to an approximate cost of $0.0625 per bird (4).

Necrotic enteritis is primarily caused by *Clostridium perfringens* (*C. perfringens*), an anaerobic gram-positive bacterium that forms spores (5). *Clostridium perfringens* is widely distributed in the environment and is commonly found in the gastrointestinal tract of various animal species, including poultry, livestock, and humans, both in healthy and diseased individuals (5, 6). Among the toxins associated with NE pathogenesis, α-toxin derived from *C. perfringens* has traditionally been identified as the primary toxin (7). However, the discovery of a novel toxin called necrotic enteritis toxin-B like (NetB) has attracted considerable attention due to its significant role in the development of NE (8). According to the established classification scheme based on toxin production, *C. perfringens* type G has emerged as a prominent causative agent of NE in poultry (9). This is attributed to its ability to produce both α-toxin and the NetB protein (9).

Necrotic enteritis in birds exhibits two distinct manifestations: clinical (acute) and subclinical (chronic) (1). The acute form is characterized by diarrhea, depression, sternal recumbency, and high flock mortality rates, which can reach up to 50% (10, 11). Pathologically, this form is associated with inflammation and extensive necrosis primarily observed in the small intestines of affected birds. In contrast, the chronic form of NE is characterized by mucosal damage in the small intestine and is marked by reduced weight gain at slaughter, decreased feed intake, and impaired growth performance (10, 11). NE typically affects birds in good body condition within the 2–6-week period, as maternal antibodies only provide protection for approximately 3 weeks (12–16). However, there have been reports of NE occurring in commercial layers older than 3 months of age (13).

Historically, antimicrobial agents were employed in chicken feed to enhance growth, modulate intestinal microbiota, and prevent the occurrence of NE (17). However, the use of these antibiotics resulted in the emergence of antibiotic resistance and posed risks to public health (18). As a response, the European Union implemented a prohibition on the inclusion of antibiotics in chicken feed in 2006, leading to a subsequent rise in NE cases (17). Consequently, alternative strategies have been implemented in poultry management to tackle NE, including the utilization of vaccination, prebiotics, probiotics, and plant-derived products (19). The reproduction of NE in experimental settings is an integral part of vaccine development studies and plays a crucial role in evaluating the effectiveness of preventive measures against NE. Several critical factors contribute to the successful induction of NE, including the careful selection of a virulent strain of *C. perfringens* capable of reproducing NE, precise preparation of the challenge inoculum, meticulous timing and route of infectious challenge inoculation, and consideration of relevant predisposing factors. Hence, it is imperative to thoroughly review the impact of these factors on NE reproduction and make informed decisions regarding their incorporation in the design of experimental NE disease. In the current study, we conducted a review of the methodologies employed for inducing NE disease during challenge experiments. Our objective was to provide a comprehensive summary of the experimental designs used to reproduce NE in related studies conducted between 2004 and 2023. The review highlights the need for further investigation and research in areas where ambiguity exists.

## 2 Selection of virulent challenge strains

Being the causative agent of avian NE, *C. perfringens* is recognized for its capacity to generate a diverse array of toxins (20, 21). The α-toxin from *C. perfringens* has traditionally been acknowledged as the principal virulence factor that elicits NE in birds (22). In the post-antibiotic era, extensive endeavors were directed toward developing effective vaccines for this particular toxin, with the aim of managing NE. Nonetheless, the importance of α-toxin in the development of NE was called into question following the revelation that pathogenicity persisted in chickens despite the absence of this toxin in a *C. perfringens* mutant (7). According to these findings, it was established that there were supplementary elements, apart from the α-toxin, that potentially contribute to the initiation of NE in birds. In a critical study, a novel protein, NetB, was isolated from a *C. perfringens* strain found in a bird afflicted with NE (22). Subsequently, it was discovered that *C. perfringens netb* knockout mutants were unable to induce NE, leading to the hypothesis that NetB may be the primary virulence factor involved in the pathogenesis of NE (22).

**Figure 1:**
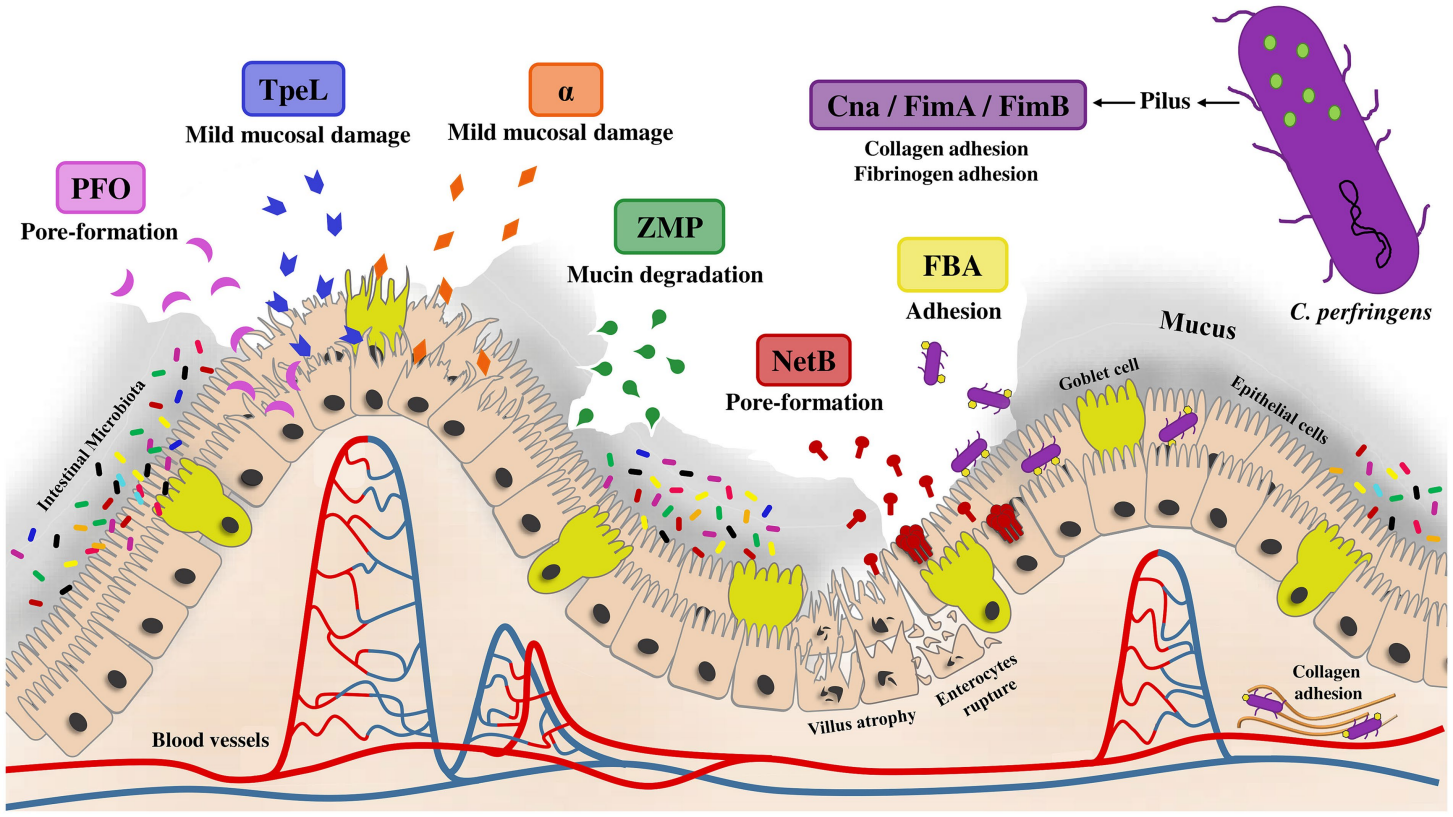
The role of *Clostridium perfringens* antigens in NE pathogenesis. Numerous antigens and toxins produced by *C. perfringens* may play a role in the pathogenesis of NE, while their presence is not crucial for the development of NE. NetB serves as the principal toxin responsible for NE by forming pores and penetrating the intestinal mucosa. Other toxins, such as α-toxin and PFO, might play distinct roles in the pathogenesis of NE by causing mucosal degradation and forming pores in epithelial cells, respectively. Other antigens involved in NE pathogenesis may include those responsible for mucus-covered epithelial cell degradation (ZMP), *C. perfringens* attachment to intestinal lining cells (FBA), collagen interaction (Cna, FimA, and FimB), and mucosal damage leading to tissue degradation (TpeL).

To effectively evaluate the efficacy of alternative approaches to combat NE in the post antibiotic era, it is imperative to induce the NE disease in experimental animal models. This highlights the importance of utilizing virulent strains of *C. perfringens* in infectious challenge studies to accurately induce the disease as it occurs in field conditions and to facilitate accurate assessments of the efficacy of the preventive measures. Since NetB toxin is the primary antigen responsible for causing NE (22), it becomes crucial to utilize *C. perfringens* strains that demonstrate a positive presence of the *netb* gene to reproduce NE *in vivo*. The development of NE-associated gut lesions is unattainable in the absence of *netb* gene. Although the presence of NetB as the major causative agent is critical and enough for reproduction of NE, other virulence factors from *C. perfringens* may contribute synergistically to intensify the severity of NE. In this regard, the co-presence of TpeL, another toxin from *C. perfringens*, along with NetB, has been demonstrated to potentially result in more severe intestinal lesions (23). Additional toxins and antigens derived from *C. perfringens* might potentially influence the pathogenesis of NE such as fructose 1, 6-biphosphate aldolase (FBA) (24), zinc metallopeptidase (ZMP) (25), perfringolysin O (PFO) (26), and pilin structural subunits (Cna, FimA, and FimB) (27, 28) (Figure 1). Despite the presence of these toxins and antigens, their involvement is not crucial for the experimental induction of NE.

**Figure 2:**
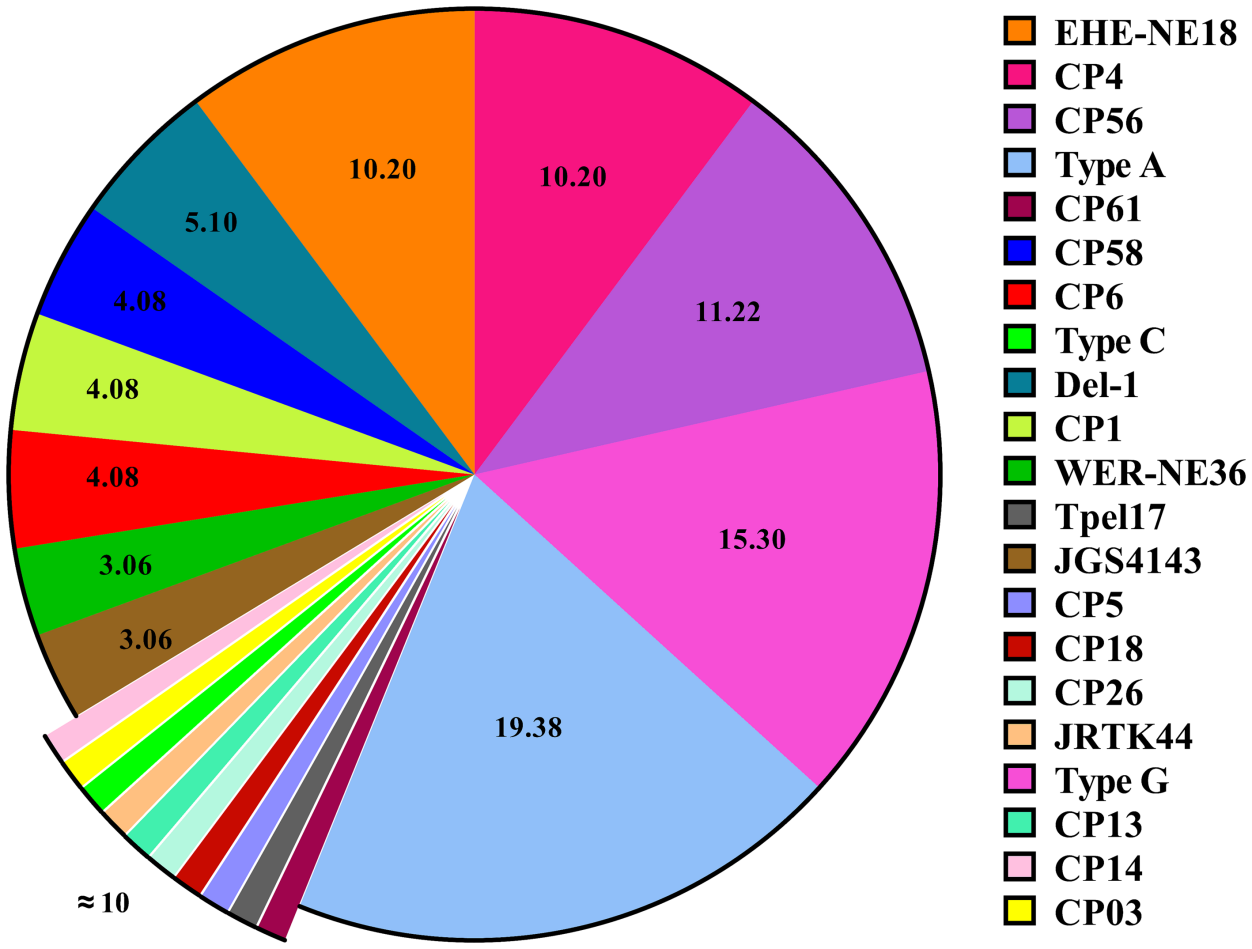
Virulent strains of *Clostridium perfringens* employed in experimental challenges to induce NE. Various strains of *C. perfringens* have been utilized for inducing *in vivo* NE infection in birds, with strains 56, 4, and EHE-NE18 being the most frequently employed in experimental challenges. A total of 93 studies that included at least one NE challenge experiment were considered in the analysis. The numerical values represent the relative proportion or percentage of studies in relation to the overall number of studies conducted. In certain studies, multiple strains were employed in the experimental design. The chart was generated using GraphPad Prism 9.0 (Graph-Pad Software, San Diego, CA, United States).

The comprehensive overview of the challenge experiments conducted to induce NE in birds is represented in Table 1. Diverse virulent strains of *C. perfringens* have been demonstrated to induce NE in broiler chickens during experimental challenge studies (Figure 2). Among these strains, *C. perfringens* strains CP56, CP4, and EHE-NE18 are the most commonly used strains for NE experimental induction, followed by Del-1, CP58, CP1, and CP6. Other less frequently utilized strains include WER-NE36 and JGS4143. A great number of studies utilized *C. perfringens* type A strains derived from NE-affected flocks to induce NE infection in their challenge experiment. However, it is of utmost importance to acknowledge that strains exhibiting *netb* positivity were categorized under *C. perfringens* type A prior to the implementation of the novel *C. perfringens* classification system based on toxin types (9). Since numerous vaccine studies were conducted prior to the introduction of the current scheme, these investigations documented the utilization of *C. perfringens* type A strains in their experimental paradigm. Now we know that these strains belong to *C. perfringens* type G. The continued utilization of type A strains in recent studies is attributed to the prevailing absence of reclassification of these *netb*-positive strains within the G type category, as mandated by the updated toxinotyping scheme. In addition, several researchers employed unidentified strains of *C. perfringens* type G, which were isolated from afflicted birds during an outbreak of NE within a flock.

**Table 1:** Summary of the *C. perfringens* challenge experiments used in NE vaccine studies

Challenge strain	Route of administration (CFU/ml or culture/feed Ratio (v/w))	Experiment program No. of days (days of age / No. of daily inoculations)	Lesion score ^1^	Scoring system	Predisposing factor(s)	Reference
CP4	In-feed (2:1)	5 CD ^2^ (23-27 / 2)	2.09	0-4	Protein-rich diet (20% to 28%) ^3^ Starvation (20 h)	(29)
	In-feed (2:1)	**Mild** 3 CD (28-30 / 2)	1.55	0-5	Wheat-based diet Protein-rich diet (20% to 28%) Starvation (20 h)	(30)
		**Moderate** 5 CD (28-32 / 2)	2.20			
		**Severe** 5 CD (28-32 / 2)	2.68			
	In-feed (2:1)	5 CD (29-33 / 2)	2.25	0-5	Protein-rich diet (20% to 28%)	(31)
	In-feed (2:1)	5 CD (29-33 / 2)	1.28	0-5	Wheat-based diet Protein-rich diet (20% to 28%) Starvation (24 h)	(32)
	In-feed (2:1)	5 CD (23-27 / 2)	2.25	0-5	Wheat-based diet Protein-rich diet (21% to 28%) Starvation (20 h)	(33)
			2.76		Turkey-based diet Protein-rich diet (21% to 28%) Starvation (20 h)	
	Oral gavage + In-feed (1:1)	5 CD (28-32 / 2)	4.42	0-6	Wheat-based diet Protein-rich diet (21% to 28%) Starvation (20 h)	(34)
	Oral gavage + In-feed (1:1)	5 CD (28-32 / 2)	4.21	0-6	Wheat-based diet Protein-rich diet (21% to 28%) Starvation (15 h)	(35)
	Oral gavage + In-feed (1:1)	5 CD (28-32 / 2)	4	0-6	Wheat-based diet Protein-rich diet (21% to 28%) Starvation (15 h)	(24)
	Oral gavage (10^8^)	3 CD (18-20 / 1)	1.80	0-6	*E. maxima* (day 14)	(36)
	Oral gavage (10^8^)	3 CD (19-21 / 1)	0.9	0-3	*E. maxima* (day 14)	(37)
JGS4143	Oral gavage + In-feed (1:1)	4 CD	3.3 ^4^	0-5	Wheat-based diet Protein-rich diet	(38)
	In-feed (3:4)	4 CD (25-28 / 1)	2.37	0-4	Protein-rich diet (20% to 28%)	(39)
	Oral gavage (10^9^)	3 CD (24-26 / 1)	1.8	0-4	Starvation (12 h)	(40)
CP56	Oral gavage (4 × 10^8^)	4 CD (17-20 / 1)	ND ^5^	0-6	Wheat/rye-based diet Protein-rich diet (to 30%) IBD vaccine (day 16) Anticoccidial vaccine (day 18)	(41)
	Oral gavage (4 × 10^8^)	4 CD (17-20 / 1)	1.56	0-6	Wheat/rye-based diet Protein-rich diet (to 30%) IBD vaccine (day 16) Anticoccidial vaccine (day 18)	(42)
		4 CD (17-20 / 3)	1.73			
		4 CD (17-20 / 1)	3.46			
		4 CD (17-20 / 1)	1.51			
CP56	Oral gavage (4 × 10^8^)	4 CD (17-20 / 3)	2.19	0-6	Wheat/rye-based diet Protein-rich diet IBD vaccine (day 16) Anticoccidial vaccine (day 18)	(43)
			2.98		Wheat/rye-based diet Protein-rich diet IBD vaccine (day 16) Anticoccidial vaccine (day 18) Heat stress (35 ºC / day 17-35)	
	Oral gavage (4 × 10^8^)	4 CD (17-20 / 1)	1.07	0-6	Wheat/rye-based diet Protein-rich diet (to 30%) IBD vaccine (day 16) Anticoccidial vaccine (day 18/20)	(44)
	Oral gavage (4 × 10^8^)	**Mild** 4 CD (17-20 / 1)	0.75	0-6	Wheat/rye-based diet Protein-rich diet (to 30%) IBD vaccine (day 16) Anticoccidial vaccine (day 18)	(45)
	In-feed (3:4)	**Severe** 4 CD (19-22 / 2)	1.70			
	Oral gavage (4 × 10^8^)	4 CD (17-20 / 1)	0.45	0-6	Wheat-based diet Protein-rich diet (to 30%) IBD vaccine (day 16)	(46)
			1.1		Wheat-based diet Protein-rich diet (to 30%) IBD vaccine (day 16) Fusarium mycotoxin deoxynivalenol	
	Oral gavage (4 × 10^8^)	4 CD (17-20 / 1)	0.65	0-6	Wheat-based diet Protein-rich diet (to 30%) IBD vaccine (day 16)	(47)
			0.77		Wheat-based diet Protein-rich diet (to 30%) IBD vaccine (day 16) Fumonisins mycotoxins		Oral gavage (4 × 10^8^)	4 CD (17-20 / 3)	2.18	0-6	Wheat/rye-based diet Protein-rich diet IBD vaccine (day 16) Anticoccidial vaccine (day 18)	(48)
3.20	Wheat/rye-based diet Protein-rich diet IBD vaccine (day 16) Anticoccidial vaccine (day 18) High Stock density											
Oral gavage (4 × 10^8^)	4 CD (17-20 / 3)	2.19	0-6	Wheat/rye-based diet Protein-rich diet IBD vaccine (day 16) Anticoccidial vaccine (day 18)	(49)
		3.79		Wheat/rye-based diet Protein-rich diet IBD vaccine (day 16) Anticoccidial vaccine (day 18) Cold stress (15 ºC)		
Oral gavage (4 × 10^8^)	4 CD (17-20 / 3)	4.6	0-6	Wheat/rye-based diet Protein-rich diet IBD vaccine (day 16) *E. maxima* (day 18)	(50)
Oral gavage (4 × 10^8^)	4 CD (17-20 / 3)	2.19	0-6	Wheat/rye-based diet Protein-rich diet IBD vaccine (day 16) Anticoccidial vaccine (day 18)	(51)
CP58			1.26	0-5	Protein-rich diet (20% to 40%) Starvation (18 h)	(52)
	Oral gavage (2 × 10^7^-10^8^) + In-feed (1:10)	5 CD (28-32 / 2)	3.33	0-5	Wheat-based diet Protein-rich diet (20% to 40%) Starvation (20 h)	(53)
	Oral gavage (10^9^) + In-feed (1:2)	4 CD (30-33 / 2)	3	0-6	Wheat-based diet Protein-rich diet (21.5% to 48%) Starvation (12 h)	(54)
	Oral gavage (10^9^) + In-feed (1:2)	4 CD (30-33 / 2)	3	0-6	Wheat-based diet Protein-rich diet (21.5% to 48%) Starvation (12 h)	(55)
WER-NE36	In-feed (3:4)	**Severe** 2 CD (26-27 / 2)	2.8	0-6	Wheat-based diet Protein-rich diet (20% to 50%)	(56)
	In-feed (4:3)	4 CD (19-22 / 1)	4.25	0-6	Wheat-based diet Protein-rich diet (20% to 50%)	(57)
	Oral gavage (10^8^)	2 CD (14-15 / 1)	1	0-4	*Eimeria spp.* (day 9)	(58)
EHE-NE18	In-feed (3:4)	2 CD (21-22 / 2)	2.6		0-6	Wheat-based diet Protein-rich diet (20% to 50%)	(59)
		2 CD (14-15 / 2)	3				
	Oral gavage (10^9^-10^10^) + In-feed (1:10)	**Mild** 2 CD (24-25 / 2)	1.9		0-6	Wheat-based diet Protein-rich diet (20% to 50%)	(56)
	In-feed (3:4)	**Moderate** 2 CD (26-27 / 2)	2				
	Oral gavage (10^8^)	2 CD (14-15 / 1)	0.83		0-4	*Eimeria spp.* (day 9)	(58)
	Oral gavage (10^8^-10^9^)	Day 14 / 1	1.25		0-4	*Eimeria spp.* (day 9)	(60)
	Oral gavage (10^8^)	2 CD (14-15 / 1)	**Exp. 1**	1.5	0-4	Anticoccidial vaccine (day 9)	(61)
			**Exp. 2**	1.8			
	Oral gavage (10^8^-10^9^)	2 CD (14-15 / 1)	1.3		0-4	*Eimeria spp.*	(62)
			0.45			Protein-rich diet (to 25%)	
			1.4			*Eimeria spp.* Protein-rich diet (to 25%)	
	Oral gavage (10^8^-10^9^)	2 CD (14-15 / 1)	1.3		0-4	*Eimeria spp.* (day 9)	(63)
			0.45			Protein-rich diet (to 25%)	
			1.4			*Eimeria spp.* (day 9) Protein-rich diet (to 25%)	
	Oral gavage (10^8^)	2 CD (14-15 / 1)	0.8		0-6	*Eimeria spp.* (day 9)	(64)
	Oral gavage (10^8^)	2 CD (14-15 / 1)	0.8		0-6	*Eimeria spp.* (day 9)	(65)
	Oral gavage (10^8^)	2 CD (14-15 / 1)	0.68		0-6	Anticoccidial vaccine (day 9)	(66)
Del-1	Oral gavage (10^9^)	Day 18	3		0-4	Protein-rich diet (17% to 24%) *E. maxima* strain 41A	(67)
	Oral gavage (10^9^)	Day 18	2.5		0-4	Protein-rich diet (18% to 24%) *E. maxima* (day 14)	(68)
	Oral gavage (10^9^)	Day 21 / 1	3.17		0-4	Protein-rich diet (18% to 24%) *E. maxima* (day 18)	(69)
		Day 18 / 1	0.87			Protein-rich diet (21% to 24%) *E. maxima* (day 14)	
	Oral gavage (10^9^)	Day 18	**Cobb**	≈ 3.5	0-4	Protein-rich diet (17% to 24%) *E. maxima* (day 14)	(70)
			**Ross**	≈ 3.3			
			**Hubbard**	≈ 2.8			
	Oral gavage (10^9^)	Day 18	2.9		0-4	Protein-rich diet (17% to 24%) *E. maxima* (day 14)	(71)
CP61	Oral gavage (4 × 10^8^)	4 CD (17-20 / 1)	ND		0-6	Wheat/rye-based diet Protein-rich diet (to 30%) IBD vaccine (day 16) Anticoccidial vaccine (day 18)	(41)
CP1	In-feed (2:1)	5 CD (29-33 / 1)	1.63		0-5	Wheat-based diet Protein-rich diet (20% to 28%) Starvation (24 h)	(32)
	In-feed (2:1)	2 CD (28-29 / 2)	2.87		0-6	Protein-rich diet (20% to 28%) Starvation (24 h)	(72)
		2 CD (26-27 / 2)	3.66				
	Oral gavage (2.5 × 10^8^)	3 CD (14-16 / 1)	0.95		0-6	**-**	(73)
		4 CD (15-18 / 1)	1.16			Wheat-based diet Protein-rich diet (18% to 60%)	
		4 CD (15-18 / 1)	1.58			Wheat-based diet Protein-rich diet (18% to 60%) Anticoccidial vaccine (day 10)	
	Oral gavage (1-2 × 10^8^)	2 CD (12-13 / 1)	0.83		0-6	-	(74)
			2.66			Corticosterone in feed (day 11)	
CP6	Oral gavage (10^8^)	3 CD (19-21 / 1)	0.9		0-3	*E. maxima* (day 14)	(37)
	Oral gavage (10^8^)	2 CD (19-20 / 1)	**Trial 1**	1.27	0-3	*E. maxima* (day 14)	(75)
			**Trial 2**	1			
	In-feed	3 CD (13-15 / 1)	**Trial 3**	0.92		-	
	Oral gavage (10^8^)	3 CD (18-20 / 1)	0.37		0-3	*E. maxima* (day 14) Starvation (2-3 h)	(76)
	Oral gavage (10^8^)	3 CD (19-21 / 1)	1.11		0-3	*E. maxima* (day 14)	(77)
CP13	Oral gavage (107)	5 CD (15-19 / 2)	2.89		0-3	Wheat-based diet Anticoccidial vaccine (day 13)	(78)	
			3.45			Wheat-based diet IBD vaccine (day 14)		
			1.67			Wheat-based diet Anticoccidial vaccine (day 13) IBD vaccine (day 14)	
CP14			2.78			Wheat-based diet Anticoccidial vaccine (day 13)		
			2.56			Wheat-based diet IBD vaccine (day 14)		
			1.78			Wheat-based diet Anticoccidial vaccine (day 13) IBD vaccine (day 14)	
CP03						3.34			Wheat-based diet Anticoccidial vaccine (day 13)		
			2.23			Wheat-based diet IBD vaccine (day 14)		
			2.43			Wheat-based diet Anticoccidial vaccine (day 13) IBD vaccine (day 14)	
Type A	In-feed (2:1)	3 CD (35-37 / 1)	ND		0-4	ND	(79)
	In-drinking water (1:2)						
	Oral gavage (4 × 10^8^)	2 CD (14-15 / 1)	3		0-6	ND	(80)
	Oral gavage (10^8^)	2 CD (23-24 / 1)	1.97		0-4	*E. maxima* (day 18) *Salmonella* Typhimurium (day 1)	(81)
	Oral gavage (2.2 × 10^8^)	5 CD (18-22 / 1)	ND		ND	Anticoccidial vaccine (day 15)	(82)
	Oral gavage (10^8^)	3 CD (19-21 / 1)	3.8 ^4^		0-6	Anticoccidial vaccine (day 12)	(83)
	Oral gavage (2.2 × 10^7^)	3 CD (18-20 / 2)	2.94		0-4	Anticoccidial vaccine (day 14) IBD vaccine (day 14)	(84)
	Oral gavage (10^8^)	Day 14 / 1	**Exp. 1**	2.1	0-4	*E. maxima* (day 9)	(85)
			**Exp. 2**	1.6			
	Oral gavage (6-8 × 10^8^)	4 CD (18-21 / 3)	3		0-4	IBD vaccine (day 16) Anticoccidial vaccine (day 19)	(86)
	Oral gavage (10^7^)	3 CD (17-19 / 2)	1.25		0-4	Wheat-based diet	(87)
			1.50			Wheat-based diet Anticoccidial vaccine (day 14)	
			1.60			Wheat-based diet IBD vaccine (day 14)	
			1.90			Wheat-based diet Anticoccidial vaccine (day 14) IBD vaccine (day 14)	
	Oral gavage (10^8^)	2 CD (22-23 / 1)	2.10		0-4	*E. maxima* (day 18)	(88)
			3.30			*E. maxima* (day 18) *Salmonella* Typhimurium (day 1)	
			2.20			*E. maxima* (day 18) *Salmonella* Typhimurium (day 17)		
	Oral gavage (2.2 × 10^8^)	3 CD (18-20 / 1)	1.33			*Eimeria spp.* (day 14) Starvation (8 h)	(89)
	Oral gavage (4 × 10^8^)	3 CD (26-28 / 1)	1.86		0-3	Anticoccidial vaccine (day 23)	(90)
	Oral gavage (10^9^)	7 CD (17-23 / 1)	1.13		0-4	*E. maxima* (day 12)	(91)
	Oral gavage (2 × 10^8^)	7 CD (15-21 / 1)	2.25		0-4	ND	(92)
	Oral gavage (2 × 10^8^)	4 CD (18-21 / 1)	1.67		0-4	Anticoccidial vaccine (day 14) Starvation (overnight)	(93)
	Oral gavage (3 × 10^8^)	3 CD (18-20 / 1)	2.64		0-6	Anticoccidial vaccine (day 14)	(94)
	Oral gavage (5 × 10^8^)	3 CD (15-17 / 1)	2.80		0-5	*E. acervolina* (day 7)	(95)
	Oral gavage (3.5 × 10^8^)	3 CD (14-16 / 1)	3.18		0-4	*Eimeria spp.* (day 9) Protein-rich diet	(96)
	Oral gavage (10^7^)	3 CD (17-19 / 1)	3.30		0-4	Anticoccidial vaccine (day 14)	(97)
Type C	In-feed (2:1)	3 CD (35-37 / 1)	ND		0-4	ND	(79)
	In-drinking water (1:2)						
TpeL17	Oral gavage (10^9^)	4 CD (23-26 / 1)	3		0-4	Protein-rich diet (16% to 24%) *E. maxima*	(98)
CP5	Intracloacal (5.8-8 × 10^8^)	4 CD (18-21 / 2)	0.66		0-6	Protein-rich diet (20% to 30%)	(99)
CP18			1.07				
CP26			1.5				
JRTK44	Oral gavage (2 × 10^8^)	Day 15 / 1	1.97		0-4	Corn-based diet Anticoccidial vaccine (day 11)	(100)
			0.57			Wheat-based diet	
			2.01			Wheat-based diet Anticoccidial vaccine (day 11)	
Other type G isolates ^6^	Oral gavage (2.5 × 10^8^)	Day 20 / 1	2.16		0-4	Anticoccidial vaccine (day 15)	(101)
	Oral gavage (3 × 10^8^)	4 CD (17-20 / 3)	2.07		0-6	Wheat-based diet Protein-rich diet	(102)
			5.07			Wheat-based diet Protein-rich diet Anticoccidial vaccine (day 18)	
	Drinking water (10^8^)	2 CD (19-20 / 1)	0.5		0-3	*E. maxima* (day 14) Starvation (4 h)	(103)
	In-feed (2:5)	3 CD (18-20 / 1)	1.2			Starvation (4 h)	
	Oral gavage (10^8^)	3 CD (19-21 / 1)	1.29			*E. maxima* (day 14)	
	In-feed (4:3)	3 CD (21-23 / 1)	4.3		0-6	Wheat-based diet Protein-rich diet (20% to 50%)	(104)
	Oral gavage (10^8^)	2 CD (19-20 / 1)	0.8		0-4	*E. maxima* (day 14)	(105)
	Oral gavage (10^9^)	Day 32 / 1	3		0-4	*E. brunetti* *E. tenella*	(106)
	Oral gavage (2.6 × 10^8^) + In-feed (1:36)	3 CD (17-19 / 2)	**Exp. 1**	3.8	0-4	IBD vaccine (day 14) Protein-rich diet Starvation (24 h)	(107)
			**Exp. 2**	3.7			
	Oral gavage (10^8^)	3 CD (19-21 / 1)	1.46		0-3	*E. maxima* (day 14)	(108)
	Oral gavage (10^9^)	2 CD (18-19 / 1)	2.04		0-4	*E. maxima* (day 13) *Salmonella* Typhimurium (day 1)	(109)
	Oral gavage (10^8^)	3 CD (18-20 / 1)	1.37		0-3	*E. maxima* (day 13)	(110)
	Oral gavage (10^8^)	3 CD (17-19 / 1)	2.31			Used litter (day 4)		
	Oral gavage (10^8^)	3 CD (19-21 / 1)	3.28		0-3	*E. maxima* (day 14)	(111)
	Oral gavage (4 × 10^8^)	3 CD (19-21 / 1)	3.33		0-6	*Eimeria spp.* (day 14)	(112)
	Oral gavage (10^8^)	3 CD (19-21 / 1)	4.30		0-3	*E. maxima* (day 14)	(113)
	Oral gavage (5 × 10^8^)	3 CD (15-17 / 1)	3		0-5	*E. acervolina* (day 14)	(95)
	Oral gavage (10^8^)	2 CD (18-19 / 1)	1.8		0-4	*E. maxima* (day 14)	(114)

^1^ The highest lesion score observed through gross examination of the small intestine of challenged control birds is considered.

^2^ CD: consecutive days

^3^ The first and second values show the percentage of crude protein in the starter and grower diets, respectively.

^4^ Scoring was carried out based on lesions observed in histopathology.

^5^ ND: Not defined

^6^ Undefined strains of *C. perfringens* type G (*netb*-positive), mainly isolated from NE-affected flocks.

**Figure 3:**
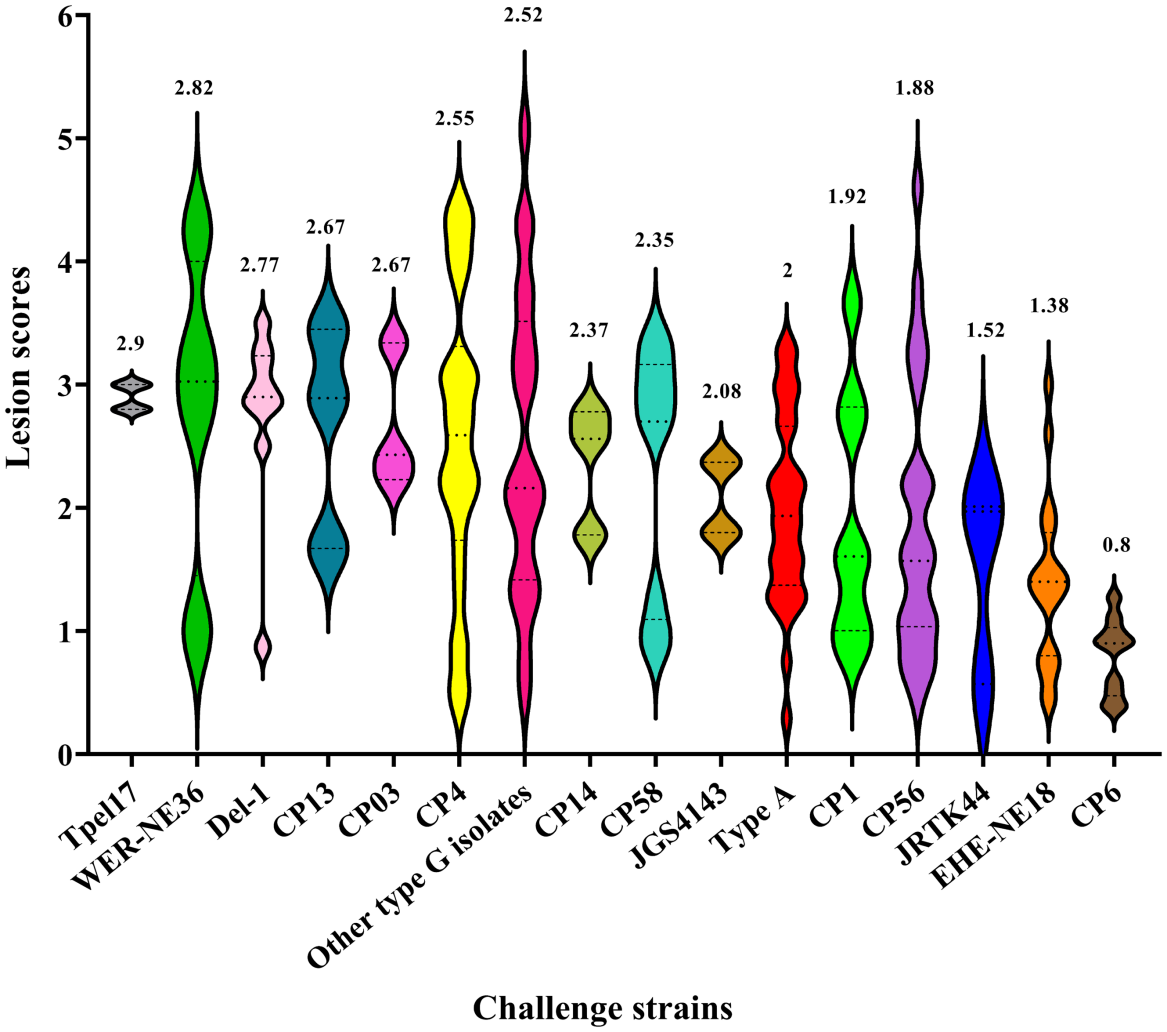
Lesion score ranges for virulent strains of *Clostridium perfringens*. The lesion scores documented in studies examining the effects of experimental NE infection in unvaccinated birds, caused by virulent strains of *C. perfringens*, have been recorded since 2004 until the present. The plotted values represent the mean NE lesion scores observed in unvaccinated birds challenged with virulent strains of *C. perfringens*. The analysis and visualization of the data were performed using GraphPad Prism 9.0 (Graph-Pad Software, San Diego, CA, United States).

Consequently, the careful selection of a virulent strain that induces more severe NE lesions plays a crucial role in accurately assessing the effectiveness and protective capacity of NE vaccines. Recent research has provided evidence that the virulent *C. perfringens* strains TpeL17, WER-NE36, Del-1, and CP13 result in more severe NE infections, as indicated by higher mean lesion scores observed in the small intestine of challenged birds (Figure 3). However, these strains have been utilized in only a limited number of vaccine studies. In contrast, strains such as CP4 have been found to induce moderate to severe lesions in the intestinal tract of challenged birds and have also been employed in a greater number of studies. The mean lesion scores of the *C. perfringens* strains used in experimental NE challenge investigations are illustrated in Figure 3.

It is important, however, to note that comparing the findings of NE infection studies is challenging due to differences in the experimental challenge infections. Numerous disparities concerning the administration and application of the virulent strains, as well as variations in the quantity and frequency of challenge inoculations, pose formidable obstacles when attempting to facilitate accurate and reliable comparisons among vaccine study results or other preventive measure investigations against NE.

## 3 NE challenge methods

### 3.1 Choice of culture media

Several culture media have been employed in the propagation of virulent strains of *C. perfringens* during challenge experiments. The selection of media has been based on a multitude of considerations, encompassing but not restricted to the simplicity of management and the efficient utilization of resources. Among the culture media employed for inducing NE *in vitro* through oral inoculation of birds or culture-infected feed or water, a fluid thioglycolate (FTG) medium is the most commonly used culture media (24, 33, 36, 37, 72, 73, 79, 87, 102, 105, 115, 116). This medium has the property of culturing under aerobic conditions due to the presence of sodium thioglycolate, a potent oxygen scavenger, in its composition. Other less commonly used media include Cooked Meat Medium (CMM) (24, 34, 38, 52, 80, 117), Brain Heart Infusion Broth (BHI) (10, 41, 42, 44–47, 53, 75, 78, 98), Tryptic Soy Broth (TSB) containing sodium thioglycolate (88, 118–120), and liver broth (85, 101). Many researchers initially cultivated *C. perfringens* in CMM before inoculating this medium into FTG medium, which is then employed for the challenge experiment by inoculating birds (24, 29, 30, 32, 34, 35, 38, 39, 53–59, 99, 104).

### 3.2 Preparation of challenge inoculum

The culture medium employed for the inoculation of birds undergoes incubation at a temperature of 37°C for a period ranging between 15 and 24 h. Research findings indicate that the 24-h culture of the FTG medium displays increased protease activity, leading to the degradation of toxins responsible for NE disease (121). Conversely, the 15-h broth cultures are observed to produce significantly higher levels of toxins in culture, which are considered essential in the formation of NE lesions, as opposed to toxins produced by vegetative *C. perfringens* in the intestinal tract post-inoculation (121). Additionally, the cultures with shorter incubation times have been shown to result in more severe lesions when compared to older cultures (24-h cultures) (29).

### 3.3 Time and route of challenge inoculation

The virulent strains utilized in challenge experiments have been administered to birds either directly through oral gavage into the crop, or indirectly by infecting feed or water with the bacterial culture (Table 1). Some researchers have employed a combination of both methods, administering the challenge inoculation through the oral route followed by an in-feed or-water challenge. In-feed inoculation possesses the characteristic of simplicity in application in contrast to the oral gavage, which induces stress associated with restraint. On the contrary, the amalgamation of broth culture and feed may result in a diminished level of the bacterium’s intended concentration (38), thereby necessitating a greater volume of broth cultures, consequently leading to an escalation in costs.

In the case of oral gavage, the challenge inoculum was composed of whole culture media containing approximately 10^8^–10^9^ bacteria per dose. The oral inoculation is usually carried out twice daily for 1 day or 5 consecutive days. A mixture of culture and feed or water at varying ratios has also been utilized twice per day for 2–5 consecutive days for the induction of NE. The ratios commonly employed for the mixture of culture and feed/water are 2:1 (v/w) (29–33, 72, 79), 3:4 (v/w) (39, 45, 56, 59), 1:1 (v/w) (24, 34, 35, 38), 2:5 (v/w) (103), 4:3 (v/w) (57, 104), and 1:2 (v/v or v/w) (54, 55, 79) for the challenge method involving the mixing of broth cultures with feed or water. Additionally, some investigators have utilized a combination of the oral route and in-feed challenge, employing cultures containing 10^8^–10^10^ colony forming unit (CFU)/dose and a mixture of culture and feed with ratios of 1:10 (v/w) (53, 56), 1:1 (v/w) (24, 34, 38), and 1:2 (v/w) (54, 55). Birds are commonly deprived of food for a duration of 2–24 h prior to the initiation of the experimental challenge. This practice serves to facilitate the process of inoculation and also establishes a state of fasting-induced stress in broiler chickens.

## 4 Predisposing factors

**Figure 4:**
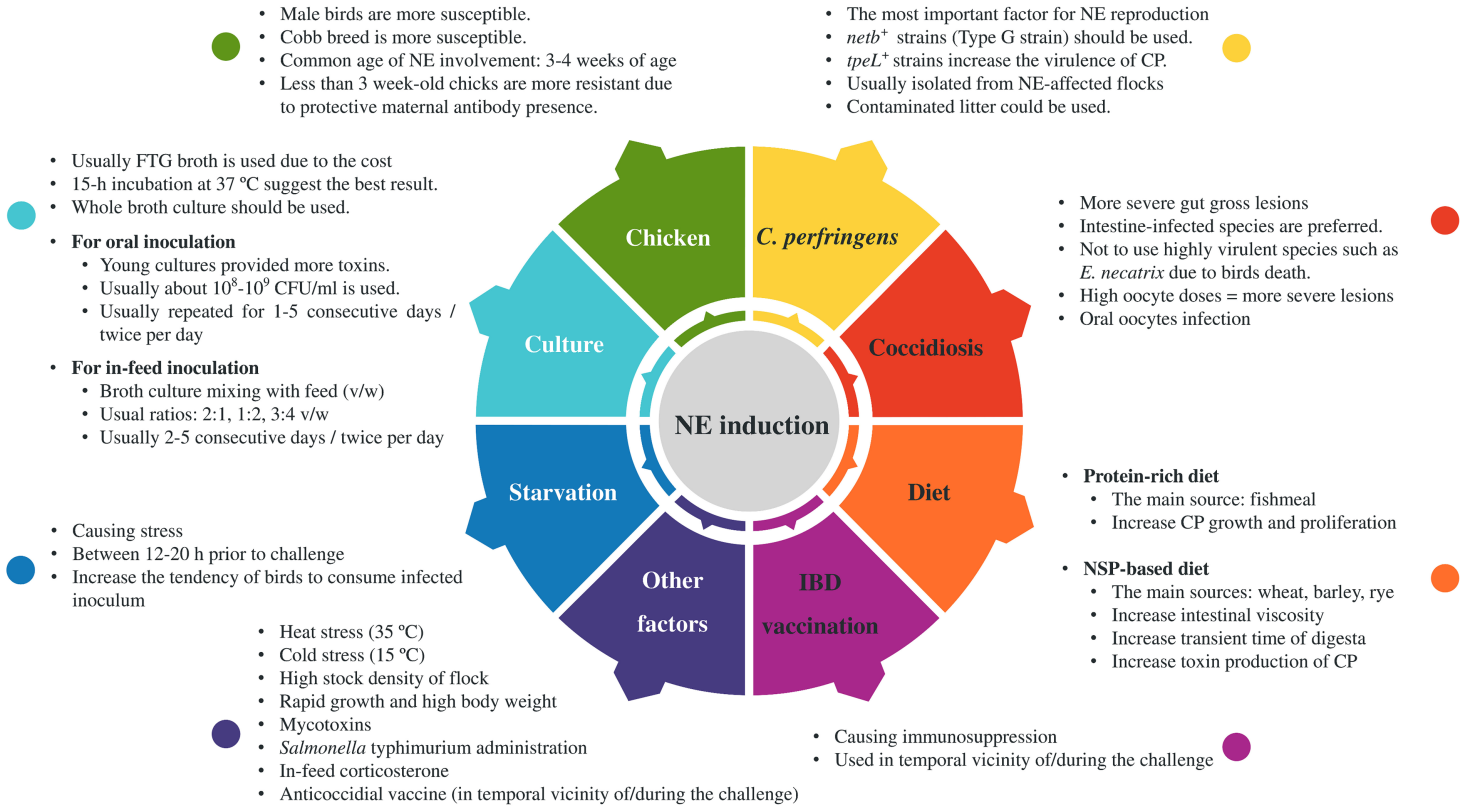
Requirements and key predisposing factors for induction of NE in birds. Successful *in vivo* induction of NE in birds necessitates careful consideration of various factors, such as the choice of virulent strains, the age and sex of the birds, and additional predisposing factors commonly associated with increased susceptibility to NE infection.

Since the experimental induction of NE requires predisposing factors, empirical studies have identified a number of factors that can increase the likelihood and severity of NE in challenged birds (Table 1). Although a few studies induced NE without using any predisposing factors (92, 122), such predisposing factors are frequently employed in vaccine studies before or during the challenge experiment as a means of augmenting the risk of NE occurrence among birds. The factors under consideration include coccidial infections, nutritional factors such as diets containing high levels of indigestible carbohydrates and crude proteins, stress, and immunosuppression resulting from vaccination with other poultry vaccines either prior to or during the challenge experiment (17, 19, 21, 123, 124). As such, these factors have been considered important variables in avian NE disease research and management programs. The predisposing factors commonly employed in NE experimental infection are illustrated in Figure 4.

**Figure 5:**
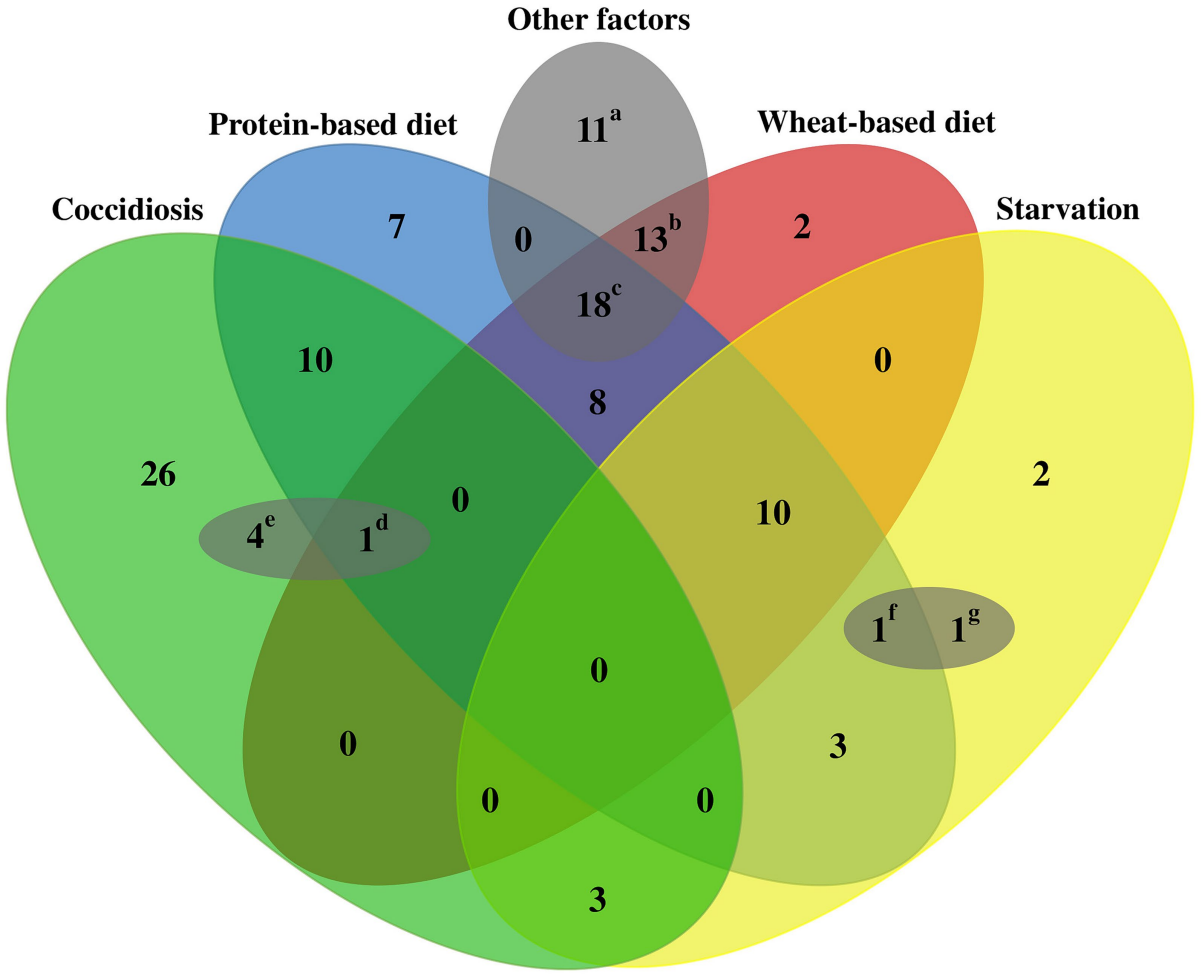
Schematic outline of common predisposing factors used for NE induction in experimental studies. A total of 120 challenge experiments were reviewed to evaluate the predisposing factors employed to induce NE in the respective infection experiments. Commonly utilized factors that predispose birds to NE include dietary factors (such as wheat-based and protein-rich diets), stress-induced starvation, and coccidiosis. In addition, several other less frequently encountered factors could also contribute to bird susceptibility, including, thermal stress variations, elevated stocking density, mycotoxin exposure, corticosterone levels, *Salmonella* species inoculation, and administration of anticoccidial and IBD vaccines in close temporal proximity to the challenge period. Each small letter includes at least one of the less frequent factors as follows: a: anticoccidial vaccines, IBD vaccines, and in-feed corticosterone; b: anticoccidial vaccines, IBD vaccines; c: anticoccidial vaccines, IBD vaccines, heat or cold stress, high stocking density, and mycotoxins; d: IBD vaccines, e: *Salmonella typhimurium* inoculation; f: IBD vaccines; and g: anticoccidial vaccines.

Of all the potential predisposing factors that have been identified for NE in birds, nutritional manipulation in chicken feed (high concentration of protein/carbohydrate), coccidiosis infection induction, and stress induction through deprivation of feed and water (starvation) are the most commonly utilized predisposing factors to induce the NE disease (Figure 5).

Coccidial infections have demonstrated a pivotal function in promoting the establishment and expansion of *C. perfringens* within the intestinal tract. It is hypothesized that the observed occurrence stems from the breakdown of the intestinal membrane, resulting in the formation of perforations in the epithelial tissue, thereby releasing plasma proteins into the intestinal lumen (21, 124). This serves as a copious nutrient source for *C. perfringens* propagation and toxin elaboration (17, 124). Coccidial infections can also induce mucogenesis, promoting the growth of *C. perfringens* (124). Several *Eimeria* species were indicated to predispose birds to NE in vaccine studies, with *E. maxima* being the most prevalent species (36, 37, 58, 67–69, 73, 75, 83–85, 98, 100, 102, 103, 105). However, it is important to note that coccidial infections are not always necessary for the occurrence of NE, as some researchers have successfully induced NE without utilizing this factor (24, 35, 52–55). Moreover, *Eimeria* infection has the potential to elicit a state of immunological stress, which may be unsuitable for conducting a vaccine study (56). This is due to the possibility of the infection-induced immune response confounding the desired immune response to the vaccine, resulting in potentially spurious or erroneous findings. As such, it is imperative to meticulously assess the immunological profile of the study participants and account for any pre-existing infections or immune perturbations when devising and executing vaccine trials.

The incidence of NE has also been revealed to be directly linked to the level of crude protein in the diet, as high protein levels can provide an optimal nutrient-rich environment for the proliferation of *C. perfringens*, thereby increasing the susceptibility of birds to NE (17, 125, 126). Fishmeal and soybean are the prevailing protein sources extensively incorporated into the diet of chickens during the challenge experiment. Additionally, cereal grains including wheat, rye, and barley could lead to an increment in the viscosity of the intestinal digesta, ultimately resulting in a prolonged bypass time (124, 127). As a result, the substrates produced by these non-starch polysaccharides could be more accessible to support the proliferation, growth, and toxin production of *C. perfringens* (100, 123, 126, 128). As such, this factor have been frequently utilized by many investigators to experimentally induce NE in birds (24, 29–35, 38, 39, 41, 42, 44, 45, 52–57, 59, 67–69, 72, 73, 98, 99, 104). The sudden implementation of these dietary modifications typically occurs during the transition from the starter diet to the grower diet, as evidenced by previous studies, thereby imposing an additional stressor on the birds involved in the experiment.

In addition to the factors mentioned above, some studies induced stress through the withdrawal of feed and water prior to the experimental infection to predispose birds to NE. The duration of the deprivation varies from a minimum of 2 h to a maximum of 24 h before the infectious challenge. Furthermore, apart from inducing stress, the state of starvation renders the process of conducting a challenge experiment more manageable, owing to the tendency of birds to consume contaminated feed post an extended period of fasting. Nonetheless, subjecting birds to intermittent periods of feed deprivation during the NE infection over consecutive days has shown the potential to mitigate the severity of gut lesions (51).

Several investigators have also employed immunosuppression induced by vaccination against several poultry diseases such as coccidiosis and infectious bursal disease (IBD) prior to the challenge experiment to predispose birds to NE (41, 42, 44, 45, 84) or inducing physiological stress using corticosterone in feed and water (74, 129, 130). Employing anticoccidial vaccines has exhibited divergent outcomes concerning NE incidence. The administration of commercial anticoccidial vaccines either immediately before or during *in vivo* NE infection for the purpose of immunosuppression may predispose birds to more pronounced NE lesions in the small intestine (78, 84, 87, 102). Conversely, the application of such vaccines during the initial day of life in chicks could potentially reduce the severity of NE lesions (50, 101, 106).

Other less common stressors demonstrated to contribute to NE-associated lesion severity. Heat and cold stresses, as environmental conditions, could play significant roles in the suppression of cellular and humoral immunity, leading to more severe gut lesions in birds (43, 49). Furthermore, elevated levels of glucocorticoids in the blood may arise as a result of heat stress and collaboratively add to the immunosuppressive impacts on heat-stressed birds (43). High body weights and fast growth also predispose birds to more severe NE disease (131). The rapid growth of birds has been elucidated to cause a transformation in the microbiota of the gastrointestinal tract and additionally resulted in the accumulation of a greater quantity of indigested or inadequately digested proteins within the gut, which consequently makes the gut a favorable environment for *C. perfringens* growth and proliferation (131, 132). This hypothesis aligns with the observation that NE typically manifests in birds exhibiting excellent body condition (11). Moreover, rearing birds in enclosures with a population that exceeds the normal stocking density (15 birds/m^2^ or 0.066 m^2^/bird) could induce stress, which suppresses the humoral immune responses, thereby increasing the likelihood of NE infection (48). Another concern that threatens overcrowded poultry farms is the elevated concentration of moisture and nitrogen emitted by the avian population, thereby diminishing the quality of the litter and creating a conducive habitat for microbial and coccidial proliferation (48). Additionally, the rivalry among birds raised in densely inhabited areas might potentially trigger heightened anxiety levels concerning nourishment intake, thus, concurrently intensifying the chances of occurring and spreading the NE infection throughout the group. Fungi may also contribute to the NE experimental model due to their ability to produce mycotoxins such as fumonisins and deoxynivalenol from Fusarium fungi (46, 47) or aflatoxin B1 from *Aspergillus flavus* (133). Oral administration of *S. typhimurium* strain in neonates is recognized as an additional contributing factor for the dependable NE induction as elucidated in some studies (81, 88, 109). The findings from these studies underscore the complex and multifaceted nature of NE pathogenesis. It is imperative to take this critical aspect into account when developing effective strategies for the prevention and control of the disease. The schematic outline of predisposing factors employed for inducing NE challenge is illustrated in Figure 5.

## 5 Other considerations

A few studies have shown the relationship between sex and breed with NE occurrence. Male birds, owing to their elevated degree of dietary intake and accelerated rate of growth, manifest a greater predisposition to NE when juxtaposed with their female counterparts (46, 134). Similarly, there have been reported breed-specific divergences in susceptibility to NE. Cobb chickens exhibit greater susceptibility to NE when contrasted with Ross and Hubbard chickens, thus manifesting more severe NE intestinal lesions subsequent to the infectious challenge experiment (70). Although the oral inoculation and/or in-feed administration of a virulent strain of *C. perfringens* is essential for the purpose of experimental NE infection, it is also feasible to induce NE without resorting to any form of inoculation through the utilization of previously contaminated/used bedding material that had been employed for NE-infected birds (110, 133, 135).

## 6 Scoring systems for NE lesions

After conducting the challenge experiment, the birds are humanely euthanized using approved methods, and subsequently undergo necropsy for additional pathological examination. For this purposes, birds are euthanized ethically through the utilization of CO2 inhalation, cervical dislocation, and electrical stunning, either on the last day of/or the day after the challenge experiment. A thorough inspection of the entire length of the small intestine is essential, and the gross pathological lesions should be evaluated using a scoring system formerly represented in the literature. The jejunum is considered to be the most impacted portion of the small intestine (58, 62, 66, 78, 84, 112, 136–140), whereas the duodenum has exhibited more pronounced lesions in certain research investigations (65, 90, 91, 141). Gross abnormalities were also apparent in the ileum and cecum, albeit to a lesser extent of involvement (2, 61–63, 65, 66, 84, 90, 91, 96, 136–138, 141). Various scoring systems have been developed to assess gross lesions associated with NE, with scales ranging from 0 to 3 (10, 12, 37, 75, 103, 142, 143), 0 to 4 (16, 29, 39, 40, 58, 67–71, 79, 85, 87, 98, 100, 101, 105, 144, 145), 0 to 5 (30–33, 38, 52, 53), and 0 to 6 (7, 22, 24, 34–36, 41, 42, 44, 45, 54–56, 59, 72, 80, 83, 102, 104, 146, 147). Although the scoring methodologies are derived from the visible lesions observed in the small intestines of birds, there have been instances where certain investigations employed scoring systems to assess the condition of the footpads in birds affected by NE (148). However, this methodology may not be entirely dependable owing to the enteropathogenic characteristics of the NE ailment. The systems that are extensively utilized for scoring NE lesions in experimental infection are depicted in Table 2.

**Table 2:** Common scoring systems used in NE studies.

Lesion score	0–6			0–5	0–4		0–3				
Reference	(7)		(121)	(30)	(144)	(85)	(149)	(150)	(12)	(10)	(151)
0	No gross lesions										
1	Thin or friable walls		Thin or friable wall or very mild and superficial generalized inflammation		Thin or friable walls	1 to 5 small white lesions (spots <1 mm in diameter)	Mild (slight mucus covering and loss of tone, thin wall, or friable)	1 to 5 small (<1-mm diameter) lesions	< 10 focal gross lesions (Focal lesion definition: their maximum extension is less than the circumference of the gut mucosa)	Focal necrosis and ulceration	Slight mucus covering and loss of tone, thin wall or friable
		Also diffuse superficial but removable fibrin									
2	Focal necrosis or ulceration (1–5 foci)		Focal necrosis or ulceration		> 5 small white lesions (spots of <1 mm in diameter) or 1 to 5 larger lesions (spots of 1 to 2 mm in diameter)		Moderate (focal necrosis or ulceration)	> 5 small lesions but fewer than 5 larger (1 to 2-mm diameter) lesions	≥ 10 focal gross lesions	Patches of necrosis 2 to 3 cm long	Focal necrosis or ulceration
		Also non-removable fibrin deposit (1–5 foci)									
3	Focal necrosis or ulceration (6–15 foci)		Large patches of necrosis		> 5 larger lesions (1 to 2 mm in diameter) or erosive zones		Marked (severe, sloughed mucosa with presence of blood in the lumen)	> 5 larger lesions and erosive zones	≥ one lesion with a maximum extension larger than the circumference of the gut mucosa	Diffuse necrosis typical of field cases	Severe, sloughed mucosa with presence of blood in the lumen
		Also non-removable fibrin deposit (6–15 foci)									
4	Focal necrosis or ulceration (16 or more foci)		Severe or extensive necrosis typical of field cases		Death with positive NE diagnoses postmortem		NA^1^				
		Also non-removable fibrin deposit (16 or more foci)									
5	Patches of necrosis 2–3 cm long		Death during the experiment with lesion scores of 4		NA		NA				
6	Diffuse necrosis typical of field cases		NA		NA		NA				

^1^Not assigned.

Owing to the variability of the systems employed for scoring NE lesions, there exists a challenge in comparing studies that adopt different scoring methods. Therefore, a standardized system for scoring NE lesions is imperative, enabling the convenient comparison of distinct groups of vaccinated birds concerning their protection against the challenge experiment. This standardized system should encompass a comprehensive range to facilitate statistical analysis, exhibit simplicity to permit the assessment of a large number of birds within a feasible time frame, maintain reproducibility among diverse observers, and also consider the severity of the disease generated under experimental conditions (121). Although it is necessary to select a broad system covering all NE lesions, the use of various antigens in immunization studies and different strains of *C. perfringens* in challenge experiments leads to variations in the gross lesions developed in NE disease (121).

## 7 Conclusion

The growing concern over restricted antibiotic usage in poultry production necessitates urgent evaluation of preventive strategies against NE. Experimental induction of NE in such studies becomes crucial for assessing the efficacy of these measures, ultimately benefiting the overall health and welfare of poultry. The development of NE is a consequence of the interplay of numerous contributing factors. These involve selecting a virulent *C. perfringens* strain, predisposing birds to NE through one or more predisposing factors, and choosing the most effective route of inoculation for optimal induction of NE disease in the challenge procedures. Selecting the ideal virulent strain containing the *netb* gene and developing an infection similar to field cases of NE is the first crucial step for experimental NE induction. Moreover, choosing among a variety of factors such as inducing coccidiosis infection, manipulating dietary protein or carbohydrates, and inducing stress conditions could raise the possibility of the NE occurrence in experimental challenges. When evaluating these factors, it is crucial to consider the potential drawbacks linked to these factors. For example, using coccidiosis infection as a predisposing factor to NE may introduce immunological alterations that could negatively impact vaccine research outcomes. Additionally, the ethical aspect of subjecting birds to stress must be considered throughout the research process. In conclusion, this review underscores the pivotal factors indispensable for the successful induction of NE. By elucidating these critical parameters, researchers can make well-informed choices when opting for the most appropriate NE experimental design.

## Author contributions

MS: Conceptualization, Data curation, Formal analysis, Investigation, Methodology, Supervision, Validation, Visualization, Writing – original draft, Writing – review & editing. MG: Supervision, Writing – review & editing.
